# Tspan9 Induces EMT and Promotes Osteosarcoma Metastasis *via* Activating FAK-Ras-ERK1/2 Pathway

**DOI:** 10.3389/fonc.2022.774988

**Published:** 2022-02-23

**Authors:** Shijie Shao, Lianhua Piao, Jiangsong Wang, Liwei Guo, Jiawen Wang, Luhui Wang, Lei Tong, Xiaofeng Yuan, Xu Han, Sheng Fang, Junke Zhu, Yimin Wang

**Affiliations:** ^1^ Department of Orthopedics, The Third Affiliated Hospital of Soochow University, Changzhou, China; ^2^ Institute of Bioinformatics and Medical Engineering, Jiangsu University of Technology, Changzhou, China

**Keywords:** osteosarcoma, Tspan9, EMT, integrin β1, metastasis, FAK-Ras-ERK1/2 pathway

## Abstract

**Object:**

At present, there are few effective treatment options available to patients suffering from osteosarcoma (OS). Clarifying the signaling pathways that govern OS oncogenesis may highlight novel approaches to treating this deadly form of cancer. Recent experimental evidence suggests that the transmembrane protein tetraspanin-9 (Tspan9) plays a role in tumor development. This study was thus formulated to assess the molecular role of Tspan9 as a regulator of OS cell metastasis.

**Methods:**

Gene expression in OS cell lines was evaluated *via* qRT-PCR, while CCK-8, colony formation, Transwell, and wound healing assays were used to explore the *in vitro* proliferative, invasive, and migratory activities of OS cells. The relationship between Tspan9 and *in vivo* OS cell metastasis was assessed by injecting these cells into the tail vein of nude mice. Interactions between the Tspan9 and integrin β1 proteins were explored through mass spectrometric and co-immunoprecipitation, and Western blotting to assess the functional mechanisms whereby Tspan9 shapes OS pathogenesis.

**Results:**

Both primary OS tumors and OS cell lines commonly exhibited Tspan9 upregulation, and the knockdown of this tetraspanin suppressed the migration, invasion, and epithelial-mesenchymal transition (EMT) activity in OS cells, whereas Tspan9 overexpression resulted in opposite phenotypes. Tumor lung metastasis were significantly impaired in mice implanted with HOS cells in which Tspan9 was downregulated as compared to mice implanted with control HOS cells. Tspan9 was also found to interact with β1 integrin and to contribute to OS metastasis *via* the amplification of integrin-mediated downstream FAK/Ras/ERK1/2 signaling pathway.

**Conclusion:**

These data suggest that Tspan9 can serve as a promising therapeutic target in OS.

## Introduction

Osteosarcoma (OS) is the most prevalent form of primary bone malignancy affecting children and adolescences, most commonly manifesting in the epiphyseal region of the proximal tibia and distal femur (1). The treatment of OS is currently primarily focused on a combination of surgical resection, adjuvant chemotherapy, and postoperative chemotherapy (2). However, as OS tumors are highly malignant and aggressive, patients generally exhibit poor 5-year survival rates, particularly in individuals with early lung metastases in whom these rates are < 30% (3). Efficacious OS treatment is hindered by the emergence of chemoresistant OS tumors (4) and by chemotherapy -induced organ damage (5). At present, no biomarkers or therapeutic targets specific for OS have been identified or leveraged in a clinical context, and there is thus a clear need to further explore the molecular mechanisms governing OS pathogenesis and metastasis in order to design more effective treatments capable of prolonging the survival of affected patients.

The tetraspanin family of conserved 4-transmembrane proteins (TM4SF) proteins each harbor a small extracellular loop (EC1), a large extracellular loop (EC2), N- and C-terminal cytoplasmic tails, and four hydrophobic transmembrane domains (TM1-4) (6). To date, 33 human tetraspanins have been identified and found to be expressed in most tissues, including Tspan8, CD63, CD82, and CD151 (7). These tetraspanins can bind with surface proteins and other molecules at the plasma membrane in tetraspanin-enriched membrane microdomains (TEMs). By interacting with integrins, receptor molecules, and other signaling intermediaries, tetraspanins can activate downstream signaling to control key physiological processes including apoptosis, adhesion, differentiation, oncogenesis, and metastatic tumor progression (8–10).

Tspan9 (NET-5, PP1057) is a tetraspanin that has been shown to promote platelet aggregation (11) and alphavirus transport (12). Prior evidence also suggests that Tspan9 can suppress the development of gastric cancer. Indeed, Li et al. (13) determined that Tspan9 was able to inhibit SGC7901 cell proliferation *via* suppressing the G1/S phase transition while simultaneously promoting MMP-9 downregulation *via* the ERK1/2 pathway to compromise cellular migratory and invasive activity. The overexpression of Tspan9 can also impair FAK-Ras-ERK signaling and EMT induction to hamper metastatic gastric cancer progression (14). Tspan9 can further enhance tumor cell 5-fluorouracil resistance *via* the inhibition of the PI3K/Akt/mTOR pathway and the induction of autophagic activity (15). Given that different TM4SF proteins play distinct roles as suppressors or promoters of oncogenesis in a context-dependent manner, however, it is impossible to reliably predict the functional role of Tspan9 as a regulator of OS tumor cell survival, migration, or metastasis. For example, both CD9 and CD82 are tetraspanins that function to primarily suppress tumor growth, whereas Tspan8 and CD151 are primarily oncogenic. However, in ovarian cancer and invasive lobular breast cancer, CD151 can bind to integrin α3β1 to regulate signaling pathways downstream of this complex, ultimately impairing tumor progression (16, 17). Tspan7 can similarly inhibit bladder cancer, liver cancer, and multiple myeloma, yet it promotes non-small cell lung cancer. No studies to date have clarified the functional importance of Tspan9 in OS, and the mechanistic role of this tetraspanin in this cancer remains to be elucidated, particularly in the context of EMT induction and tumor metastasis.

Distant tumor cell metastasis remains a major barrier to effective OS patient treatment. Epithelial-mesenchymal transition (EMT) is a process integral to metastatic progression wherein tumor cells shed their epithelial-like properties and instead acquire more motile mesenchymal characteristics. EMT induction is critical to malignant cell dissemination in the context of OS progression (18, 19), and EMT-regulating transcription factors including the zinc-finger- binding transcription factors Snai1 and Slug, zinc finger E−box−binding homeobox 1 (ZEB1), and the Twist1 and Twist2 basic helix-loop-helix (bHLH) factors have been shown to control this process in OS cells and to regulate associated metastasis (20–22). Integrins, which are heterodimeric receptors with α and β subunits that bind to the extracellular matrix, also regulate tumor malignancy. For example, the αvβ3 and β1 integrins have been linked to worse clinical outcomes and to enhanced metastasis in OS (23–25). By binding to specific ECM ligands and cell surface molecules, integrins can trigger context-appropriate intracellular signaling that can shape a variety of physiological or pathological processes (26). The focal adhesion kinase (FAK) and extracellular regulated protein kinases (ERK) enzymes are essential mediators of proliferative and migratory activity in cancer cells, and they are also directly involved in signaling downstream of integrin activation (27–29). As molecular scaffolds, tetraspanins such as CD9, CD63, CD81, CD82, CD151, and Tspan8 commonly bind to specific integrins within TEM domains. However, whether Tspan9 can interact with integrins and whether these interactions are related to OS cell aggression remain to be established.

Herein, we assessed Tspan9 expression in OS and found it to be upregulated in both OS patient tumor tissues and in OS cell lines. Through *in vitro* and *in vivo* analyses of the mechanistic importance of Tspan9 in OS, we found that it was able to not only promote EMT induction as a means of driving tumor cell metastasis, but also to interact with integrin β1 and to thereby enhance FAK-Ras-ERK1/2 signaling activity within tumor cells. Overall, our study offers new insight into the role of Tspan9 in OS and highlights its promise as a potential diagnostic biomarker and therapeutic target in this oncogenic context.

## Materials and Methods

### Microarray Data Collection

Microarray datasets related to gene expression profiles in OS samples were downloaded from the Gene Expression Omnibus (GEO) database (http://www.ncbi.nlm.nih.gov/geo). These include the GSE12865 dataset [2 normal osteoblast (OB) samples and 12 OS tissue samples], the GSE33383 dataset [11 normal mesenchymal stem cells (MSC) samples, 3 OB samples, and 84 primary OS tissue samples], and the GSE42352 dataset (11 MSC samples, 3 OB samples, and 19 primary OS cell lines), all of which were used for differential gene expression analyses.

### Cell Culture

HEK293T cells and the human hFOB1.19, HOS, U2OS, and Mg63 OS cell lines were obtained from the Chinese Academy of Cell Resource Center and were cultured in DMEM/MEM (Gibco, CA, USA) containing 10% fetal bovine serum (ScienCell, USA) and penicillin/streptomycin in a 5% CO_2_ incubator at 37°C.

### RNA Extraction and Quantitative Real-Time Polymerase Chain Reaction (qRT-PCR)

A qRT-PCR approach was used to assess change in gene expression. Briefly, a total of 1x10^6^ cells in 6-well plates were lysed to prepare RNA with a MiniBEST Universal RNA Extraction kit (Takara, Dalian, China), after which a First Strand cDNA Synthesis kit (Takara) was used to prepared cDNA based on provided directions. SYBR Premix Ex Taq (Takara) and a Step-One Plus RT-PCR System (Applied Biosystems, Shanghai, China) were used to perform qRT-PCR reactions with the following settings: 30 s at 95°C; 40 cycles of 5 s at 95°C and 34 s at 60°C. The 2^-ΔΔCt^ method was used to assess relative gene expression, with GAPDH being used for normalization. Primers used in this study are listed in **Table 1**.

**Table 1:** The siRNA and shRNA sequences used in this study.

Name		Sequences
Tspan9	Forward	AACGAGAACGCCAAGAAGGA
	Reverse	CGTTGTTCTCGGTGTGGTACA
GAPDH	Forward	ATGGAAATCCCATCACCATCTT
	Reverse	CGCCCCACTTGATTTTGG
Tspan9 siRNAs		
siTspan9#1	Sense	UGGACAAGGUGAACGAGAATT
	Antisense	UUCUCGUUCACCUUGUCCATT
siTspan9#2	Sense	CUGAAGAACGCCUGGAACATT
	Antisense	UGUUCCAGGCGUUCUUCAGTT
siTspan9#3	Sense	CCAAGAAGGACCUGAAGGATT
	Antisense	UCCUUCAGGUCCUUCUUGGTT
Negative Control (siNC)	Sense	UUCUCCGAACGUGUCACGUTT
	Antisense	ACGUGACACGUUCGGAGAATT
Tspan9 shRNAs		
shTspan9#1	5’-3’	TGGACAAGGTGAACGAGAA
shTspan9#2	5’-3’	CTGAAGAACGCCTGGAACA
Negative Control (shNC)	5’-3’	TTCTCCGAACGTGTCACGT

### Transfection

To knockdown Tspan9, OS cells were transfected with Tspan9-specific siRNA oligonucleotide duplexes (siTspan9#1, siTspan9#2, siTspan9#3) or a control siNC construct purchased from Biolino Nucleic Acid Technology Co., Ltd. Briefly, cells were grown to 30-50% confluency, at which time they were transfected with siNC or siTspan9 (100 nM) in serum-free Opti-MEM using Lipofectamine 3000 (Thermo Fisher Scientific, Inc.) based on provided directions. At 6 h post-transfection, Opti-MEM was exchanged for complete media, and knockdown efficiency was subsequently confirmed *via* qRT-PCR.

### Stable Cell Line Establishment

To stably knock down Tspan9, two shRNAs specific for this gene and one control shRNA were purchased from Shanghai GenePharma Co.,Ltd. HOS cells were then transduced with lentiviruses encoding shTspan9#1, shTspan9#2, or shNC sequences in media supplemented with 10% FBS and 8 µg/mL polybrene. At 48 h post-transduction, puromycin (2 mg/mL) was added to cells, which were then cultured for 1 week to yield stably transduced cell lined as confirmed based upon fluorescence microscopy analyses of green fluorescent protein (GFP) positivity.

A FLAG-tagged PGMLV-6946 Tspan9 expression vector was purchased from GenePharma Technology Co., Ltd (Shanghai, China). U2OS cells were transduced with prepared lentiviral particles encoding this construct, and stably transduced cells were then selected with blasticidin (2 mg/mL). In addition, stable Tspan9 overexpression and knockdown were confirmed through qRT-PCR and Western blotting. All shRNA sequences used herein are listed in **Table 1**.

### RNA-Sequencing (RNA-Seq)

Total RNA was extracted from HOS cells stably transduced with shTspan9 or shNC, and an Agilent Bioanalyzer 2100 (Agilent Technologies, Inc., USA) was used to confirm RNA integrity. An Illumina HiSeq 2000 instrument (Illumina, Inc., USA) was then used to perform deep RNA-sequencing of these samples. Differentially expressed genes (DEGs) were then identified (*P* < 0.05, and fold-change ≥ 2) and subjected to Gene Ontology (GO; http://www.geneontology.org) and KEGG (https://www.genome.jp/kegg) enrichment analyses aimed at identifying the signaling pathways whereby Tspan9 influences the development of OS.

### Viability and Proliferation Assays

A CCK-8 assay was used to evaluate cell viability. Briefly, siRNA-treated cells were seeded in 96-well plates (2000/well) in triplicate, and after 72 h, 10 uL of CCK8 solution (Dojindo Molecular Technologies, Inc., China) was added per well. After a 1 h incubation in the dark, absorbance at 450 nm was measured *via* microplate reader (Thermo Fisher Scientific, Inc.). Colony formation assays were also used to evaluate OS cell proliferation. Briefly, cells were added to 6-well plates (5000/well) and subjected to siRNA transfection. After incubation for 10-14 days, colonies were fixed for 15 min with methanol, stained for 30 min with 0.1% crystal violet, rinsed using distilled water, and colony numbers were then quantified.

### Migration and Invasion Assays

Wound healing and Transwell assays were conducted to evaluate OS cell migration. In wound healing assays, OS cells stably expressing shTspan9 or shNC were grown to 95% confluency in 6-well plates. Following starvation for 24 h in serum-free media, three horizontal and three vertical scratch wounds were generated in the cell monolayer using a sterile 1 mL pipette tip. Wounds were imaged at 0, 24, and 48 h in at least 16 fields of view per well *via* microscopy, and ImageJ was used to quantify migration as follows: migration rate (%) = [1 - (wound area at 24 h or 48 h/wound area at 0 h)] × 100%. The mean value was calculated based on 10 random fields of view. In Transwell migration assays, 2 x 10^4^ cells in 150 uL of serum-free media were added to the upper chamber of a 24-well Tranwell insert (#3464, Corning, USA), while the lower chamber was filled with 600 μL of media supplemented with 10% FBS. Following an 18 h incubation, PBS was used to wash cells two times, and they were then fixed with methanol, stained with crystal violet, and non-migratory cells were removed with a cotton swab. Transwell invasion assays were conducted *via* a similar approach using Matrigel-coated Transwell inserts (#354480, BD, USA). IN those assays, 500 μL of serum-free medium containing 1x10^5^ cells was added to the upper chamber of this insert in a 24-well plate, while the lower chamber was filled with 700 μL of media containing 10% FBS. Following a 20 h incubation, non-invasive cells were removed, while invasive cells were fixed and stained. For both Transwell assays, cells in 6 random fields of view were imaged *via* microscopy and counted.

### Western Blotting

Cells were grown to ~95% confluence in cell culture plates, at which time chilled RIPA buffer (Roche Applied Science, Penzberg, Germany) was used to extract proteins from cells for 10 min, after which samples were sonicated to facilitate lysis (Ningbo Scientz Biotechnology, Zhejiang, China). After centrifugation for 1 min at 12,000 rpm, supernatant protein levels were measured *via* BCA assay (Biyuntian Biotechnology, Shanghai, China). Next, 6-12% SDS-PAGE was used to separate protein samples prior to their transfer onto 0.45 μm PVDF membranes (Millipore, USA). Blots were then blocked for 1 h at room temperature with 5% non-fat milk in TBST, followed by overnight incubation with appropriate primary antibodies at 4°C and a subsequent 45 min incubation with secondary antibodies. Tanon Image Software v1.0 (Tanon Science and Technology Co., Ltd.) was then used to detect protein bands, with β-actin serving as a loading control. Primary antibodies specific for Tspan9 (1:300, #ab106412, Abcam), β-Catenin (1:1000, #8480, CST), N-Cadherin (1:1000, #13116, CST), FN1 (1:1000, #A12932, ABclonal), Vimentin (1:1000, #5741, CST), Snai1 (1:1000, #3879, CST), total-FAK (1:1000, #ab40794, Abcam), phospho-FAK (1:500, #AP0302, ABclonal), Ras (1:1000, #ab52939, Abcam), total-ERK1/2 (1:1000, #4695, CST), phospho-ERK1/2 (1:1000, #4370, CST), Flag (1:1000, #F7425, Sigma), and integrin β1/ITGB1 (1:500, #A2217, ABclonal) were utilized, while anti-rabbit and anti-mouse IgG (1:5000, CST) were employed as secondary antibodies.

### Immunoprecipitation (IP) Assay

U2OS cells expressing FLAG-tagged Tspan9 or control constructs were cultured in 10 cm dishes and then lysed using chilled RIPA buffer supplemented with protease inhibitors. The resultant lysates were subjected to sonication, centrifuged for 1 min at 12,000 rpm, and supernatants were transferred to a fresh tube. For IP assays, 250 μL of anti-Flag M2 agarose beads (#A2220, Sigma) were rinsed three times using PBS (2 min/wash), collected *via* centrifugation at 8,000 rpm, and combined with lysates. These samples were then incubated at 4°C for 2 h under constant agitation. Precipitates were then collected, washed three times using PBS, combined with sample loading buffer, boiled, and used for Western blotting analyses as above.

### Mass Spectrometric Analysis

HEK 293T cells were transfected with FLAG-Tspan9 or FLAG empty vector. At 48 h post-transfection, cells were immunoprecipitated with anti-FLAG M2 agarose beads. The Tspan9-interacting proteins were identified by mass spectrometry.

### Ras Inhibition

U2OS cells stably expressing the MOCK or OE-Tspan9 constructs were treated for 48 h with the Ras inhibitor Salirasib (50 μM; #HY-14754), after which they were used in assays of migratory and invasion activity. Changes in intracellular signaling proteins were assessed *via* Western blotting.

### *In Vivo* Metastasis Assay

The Animal Ethics Committee of the Third Affiliated Hospital of Soochow University approved this study. Female BALB/c nude mice (5-6 weeks old, Qinglong Mountain Animal Breeding Center, Nanjing, China) were housed in a climate-controlled animal facility (26 °C, 12h light/dark cycle) with sterile bedding, food, and water that were replaced every other day. To establish a model of murine lung metastasis, these mice received an intravenous injection of HOS cells (shNC or shTspan9#1) *via* the lateral tail vein (2.0 × 10^6^ cells/animal). After 4 weeks, all animals were euthanized and lungs were collected, immersed for 24 h in phosphate-buffered formalin, and metastases visible on the lung surface and bottom were counted by eye. Whole lungs were then embedded, cut into sections, and subjected to hematoxylin-eosin (H&E) staining. Prepared sections were imaged at 5 x and 10 x magnification.

### Statistical Analysis

SPSS v 21.0 (IBM Corp., NY, USA) was used for all statistical testing. qRT-PCR data are given as means ± standard error values, while other data are given as means ± standard deviation. *P* < 0.05 was the threshold of significance, and all studies were repeated in duplicate or triplicate.

## Results

### Human OS Tissue Samples and Cell Lines Exhibit Tspan9 Upregulation

**Figure 1:**
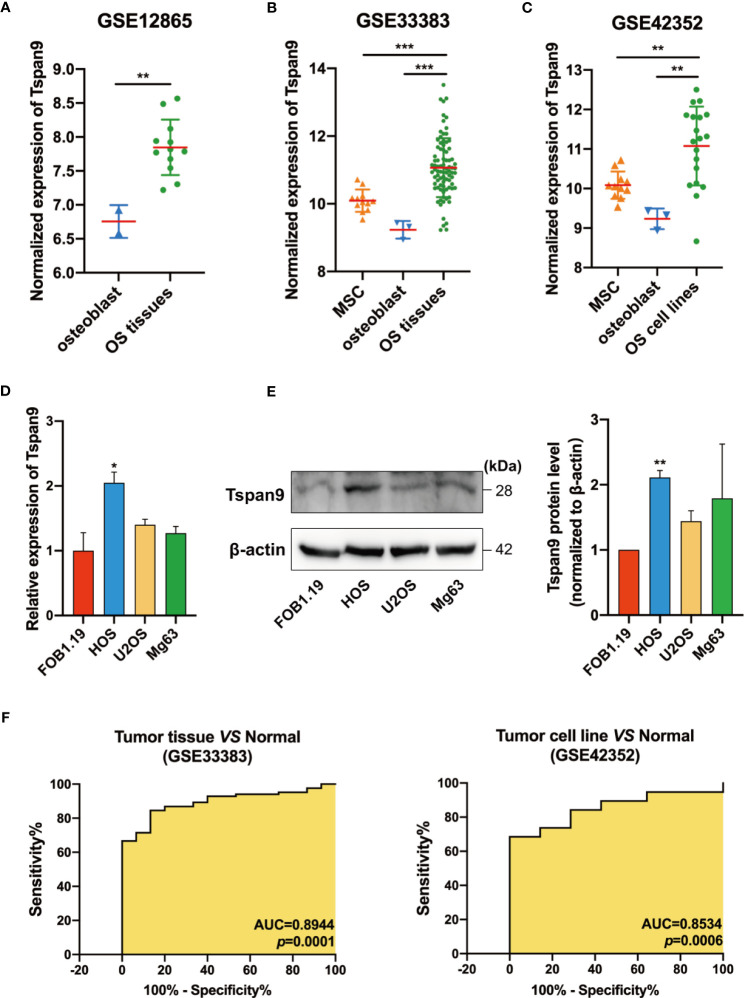
Human OS cells and tumor tissue samples exhibit Tspan9 upregulation. **(A–C)** Tspan9 mRNA levels were significantly elevated in OS tumor tissues and cell lines in the GSE12865, GSE33383, and GSE42352 datasets relative to levels in normal MSCs and OBs. **(D)** Relative Tspan9 mRNA levels were markedly increased in HOS cells relative to control hFOB1.19 cells, whereas no changes were evident in U2OS or Mg63 cells as assessed *via* qRT-PCR. GAPDH served as a normalization control. **(E)** Western blotting results revealed that Tspan9 protein expression in HOS but not U2OS and Mg63 was significantly higher compared to hFOB1.19 cells. β-actin was used as a loading control and for normalization. Data are means ± SD from two independent experiments. **(F)** ROC curves and AUC values were determined using the GSE33383 and GSE42352 datasets. *P < 0.05; **P < 0.01; ***P < 0.001; Student’s t-test. Tspan9, Tetraspanin-9; MSC, mesenchymal stem cell; OB: osteoblast; OS: osteosarcoma; GEO: Gene Expression Omnibus; qRT-PCR, quantitative reverse -transcription polymerase chain reaction; GAPDH, glyceraldehyde-3-phosphate dehydrogenase; SD, standard deviation; ROC, receiver operating characteristic; AUC, area under the curve.

We began by evaluating extant microarray datasets from the GEO database (GSE12865, GSE33383, and GSE42352) to analyze the expression of Tspan9 in human OS samples. In the GSE12865 dataset, we observed significant Tspan9 upregulation in OS tissues relative to normal osteoblast cells (OBs) (fold-change = 2.131, *P* = 0.0037) (**Figure 1A**). It was also upregulated in OS tissues compared to healthy mesenchymal stem cells (MSCs) (fold-change = 1.9651, *P* =0.0004) and OBs (fold-change = 3.5611, *P* =0.0005) according to the GSE33383 (**Figure 1B**). Similarly, in the GSE42352 dataset, Tspan9 was significantly upregulated in OS cell lines as compared to corresponding MSC (fold-change = 1.9787, *P* = 0.0037) and OB (fold-change = 3.5857, *P* = 0.0054) control samples (**Figure 1C**). We further evaluated Tspan9 expression levels in a series of human OS cell lines and found that HOS cells exhibited significantly higher Tspan9 mRNA and protein levels relative to the normal osteoblast hFOB1.19 cell line when analyzed *via* qRT-PCR (**Figure 1D**) and Western blotting assays (**Figure 1E**). Receiver operating characteristic (ROC) curves were then generated using the GSE33383 and GSE42352 datasets, and analyses of these curves indicated that Tspan9 can serve as a valuable biomarker capable of differentiating between OS tumors or cells and corresponding normal control samples (**Figure 1F**). Prior research has indicated that Tspan9 can play an anti-oncogenic function in gastric cancer. In contrast, our present results suggest that Tspan9 is upregulated in OS, suggesting that it may serve as a positive regulator of OS progression.

### Tspan9 Knockdown Suppresses the Proliferation of OS Cells

**Figure 2:**
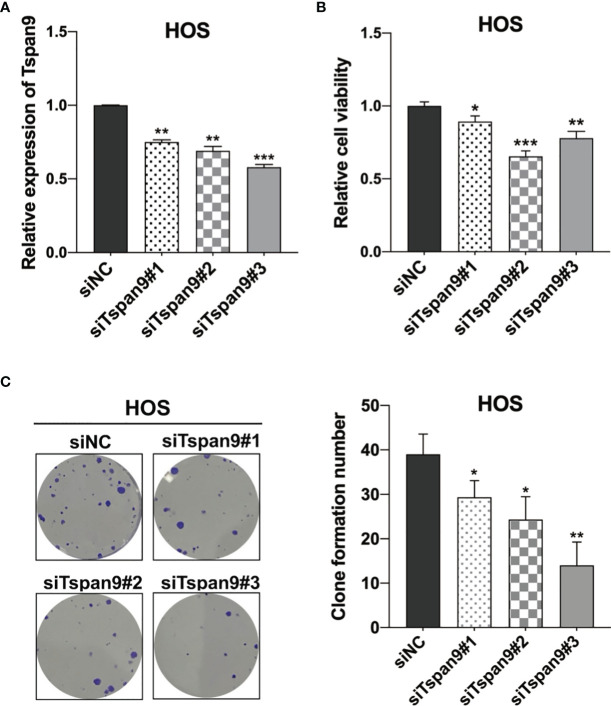
Tspan9 knockdown suppresses OS cell proliferation. **(A)** Tspan9 mRNA levels in siTspan9 cells (siTspan9#1, #2, and #3) were significantly lower than those in siNC cells, as measured *via* qRT-PCR. **(B)** The viability of HOS cells in the siNC and siTspan9 HOS cells was assessed *via* CCK-8 assay. **(C)** The impact of Tspan9 knockdown on HOS cell proliferation was measured *via* colony formation assay. Statistical results of colony formation numbers normalized to shNC were presented. *P < 0.05; **P < 0.01; ***P < 0.001; Student’s t-test. NC, negative control; si, small interfering RNA.

To clarify the functional role of Tspan9 as a regulator of OS cell proliferation, we utilized three siRNA constructs specific for this tetraspanin (siTspan9#1, siTspan9#2, siTspan9#3) to knock down its expression in HOS cells, with qRT-PCR being used to confirm Tspan9 silencing at 72 h post-transfection relative to the siNC control group (**Figure 2A**). We then conducted a CCK-8 assay to evaluate the viability of these HOS cells, and found that Tspan9 knockdown was associated with a significant decrease in HOS cell survival over a 72 h period relative to control treatment (**Figure 2B**). Colony formation assays were further conducted to explore the relationship between Tspan9 expression and the proliferation of HOS cells. On day 12 post-transfection, significantly fewer colonies were observed in the siTspan9 group relative to the siNC group (**Figure 2C**). These results thus suggest that Tspan9 may play an oncogenic role in OS cells.

### RNA-Seq-Based Identification of Downstream Tspan9 Target Genes

**Figure 3:**
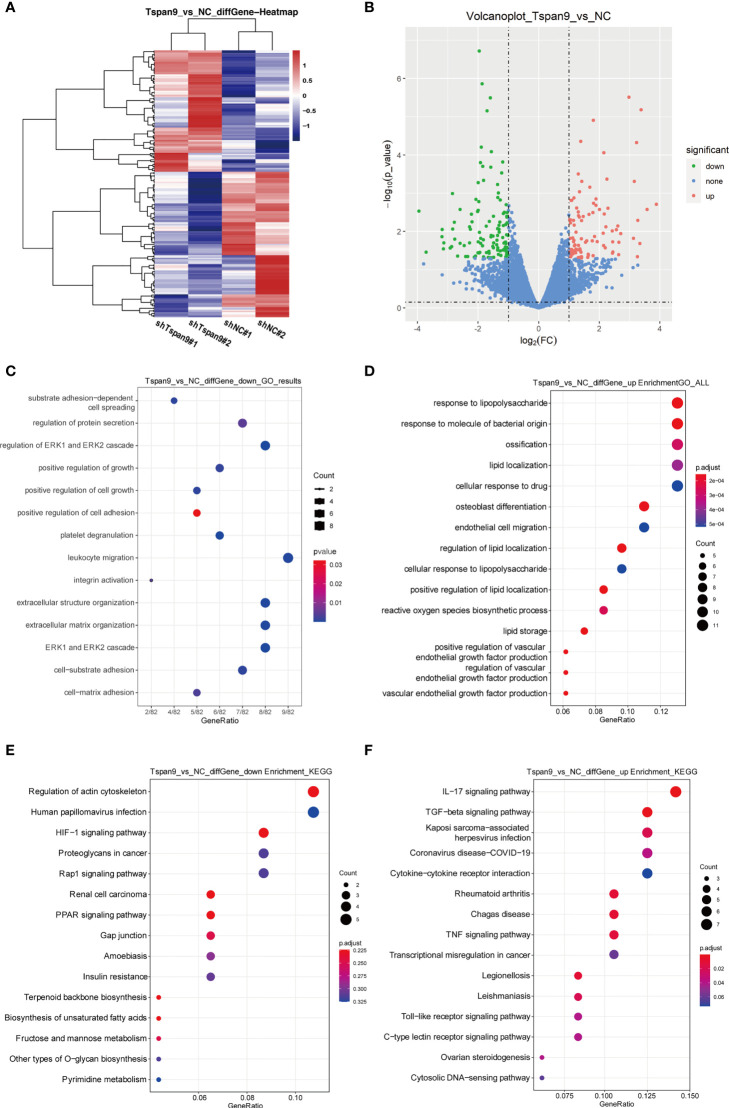
RNA-seq-mediated identification of the biological roles of Tspan9 in OS cells. **(A)** Heatmaps demonstrating DEGs identified *via* RNA-seq in HOS cells in which shTspan9 or shNC were stably expressed. **(B)** DEGs (n=211) are represented in a volcano plot, including 96 upregulated DEGs (red) and 115 downregulated DEGs (green), with DEGs having been identified using the following criteria: adjusted log fold-change ≥ 1 and *P* ≤ 0.05. **(C, D)** GO analyses of DEGs identified following Tspan9 knockdown were conducted, with top enriched biological processes, molecular functions, and cellular components being shown in a bubble chart in which darker coloration is indicative of more significant enrichment. **(E, F)** KEGG pathway enrichment analyses of identified DEGs were conducted, with the results being shown in a bubble chart in which bubble size is proportional to the number of DEGs in a given pathway, and the bubble color is indicative of P-value significance (red = significant, blue = non-significant). DEGs, differentially expressed genes; GO, Gene Ontology; KEGG, Kyoto Encyclopedia of Genes and Genomes.

To determine the targets downstream of Tspan9 in OS cells, we next conducted an RNA-seq analysis of cells in which this tetraspanin had been stably knocked down (shTspan9#1 and shTspan9#2) and compared these cells to control (shNC) cells. This approach revealed 211 genes that were differentially expressed, including 96 and 115 that were upregulated and downregulated, respectively, following Tspan9 knockdown (*P* < 0.05, fold-change ≥ 2) (**Figures 3A, B**). Notably, fibronectin 1 (FN1), which is an interstitial biomarker related to EMT induction, was found to be significantly downregulated in OS cells in which Tspan9 had been knocked down as compared to shNC cells, indicating that this tetraspanin may regulate this EMT-related gene (**Table 2**). To further elucidate the functional role of Tspan9 in this oncogenic setting, we performed a GO enrichment analysis (including cellular component, biological process, and molecular function) of these identified DEGs and found the downregulated DEGs to be enriched in the regulation of ERK1/2 cascade (GO:0070372), leukocyte migration (GO:0050900), extracellular structure organization (GO:0043062), extracellular matrix organization (GO:0030198), positive regulation of cell growth (GO:0030307) and cell adhesion (GO:0045785), and integrin activation (GO:0033622) (**Figure 3C**). Upregulated DEGs were mostly associated with the response to lipopolysaccharide (GO:0032496) and response to molecule of bacterial origin (GO:0002237) (**Figure 3D**). Besides, to explore the potential pathways enriched in DEGs, a KEGG pathway enrichment analysis of these DEGs was additionally performed (**Figure 3E**; cutoff: *P* < 0.05). we revealed these downregulated DEGs to be enriched in the HIF-1 (hsa04066), Rap1 (hsa04015), and PPAR (hsa03320) signaling pathways, and upregulated DEGs were significantly related to IL-17 signaling pathway (hsa04657), TGF-beta signaling pathway (hsa04350), and Kaposi sarcoma-associated herpesvirus infection (hsa05167) (**Figure 3F**).

**Table 2:** Eleven of 82 differential genes enriched in certain cancer metastasis-related GO processes (regulation of cell adhesion and extracellular matrix organization) based on RNA-Seq.

Gene names	Expression value -shTspan9#1	Expression value -shTspan9#2	Expression value -shNC#1	Expression value -shNC#2	Log^2^ fold change	*p*-value
*PDGFB*	2.026756025	1.684725714	2.268746532	3.530040241	-4.528380789	*p<0.05*
*TGFB2*	0.722634952	0.559786978	1.872658528	0.826405003	-3.186123662	*p*<0.05
*ITGA8*	1.023664948	1.196609075	1.978711921	1.449654294	-2.254178833	*p<0.05*
*TEK*	0.722634952	0.559786978	1.361928673	1.35788392	-2.34444508	*p*<0.05
***FN1***	**3.546946779**	**3.470766925**	**3.595974438**	**4.341457148**	**-1.998509785**	***p*<0.05**
*COL7A1*	3.551294849	3.251163207	3.958601588	4.015726844	-1.864478412	*p*<0.05
*LCP1*	1.826926919	1.735878237	1.908130846	2.513041272	-1.733824821	*p<0.05*
*COL5A1*	3.712021197	3.592540008	4.045844797	4.211975531	-1.595678527	*p<0.05*
*CITED2*	3.19830614	3.026160239	3.047824772	3.759892291	-1.37840773	*p*<0.05
*DYSF*	1.938234753	1.480605732	2.095430327	2.24800893	-1.349043777	*p*<0.05
*GAS6*	2.722014088	2.60641219	2.904711187	3.053262573	-1.051382751	*p<0.05*

The significance of row given in bold in the table is to highlight the direction of research.

### Tspan9 Enhances the *In Vitro* Migratory and Invasive Activity of OS Cells

**Figure 4:**
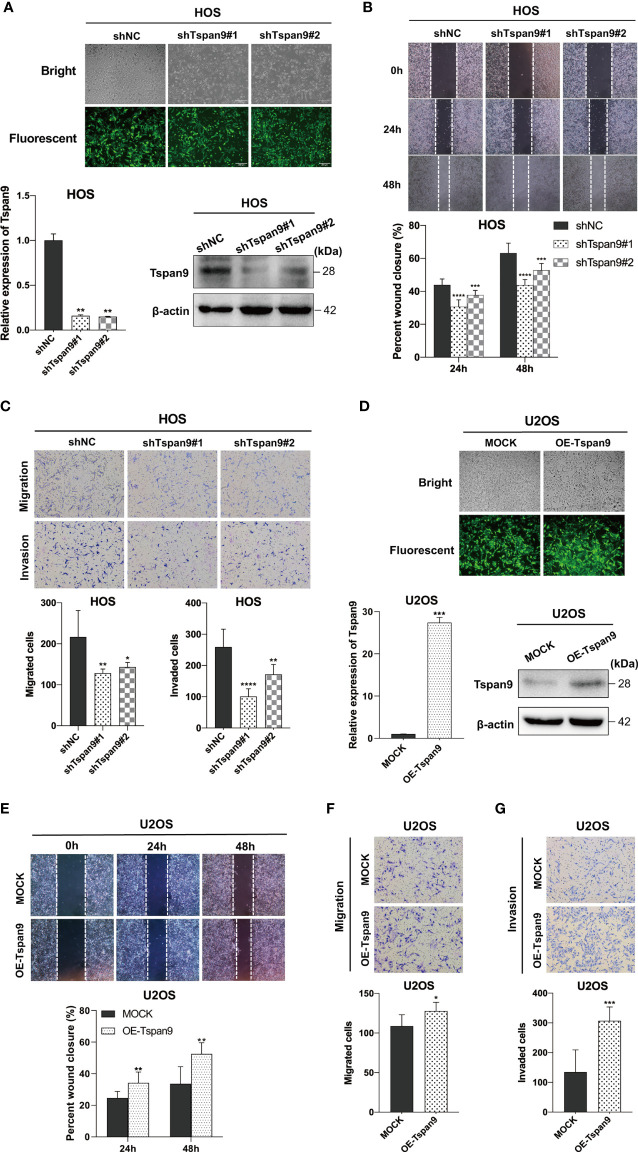
Tspan9 promotes *in vitro* OS cell migration and invasion. **(A)** GFP expression was indicative of stable lentiviral transduction of HOS cells with shTspan9 (shTspan9#1 or shTspan9#2) or shNC constructs at near 100% efficiency (fluorescent microscopy). Tspan9 knockdown efficiency was also confirmed *via* qRT-PCR (lower left panel) and Western blotting (lower right panel). **(B)** OS cell migration was assessed in a wound-healing assay using cells stably expressing shTspan9 or shNC. **(C)** The impact of Tspan9 knockdown on OS cell migration and invasion was assessed *via* a Transwell approach. **(D)** GFP expression was indicative of successful Tspan9 overexpression, as confirmed *via* qRT-PCR and Western blotting relative to Mock control. Wound-healing **(E)** and Transwell assays **(F, G)** were conducted to assess the impact of Tspan9 on the migratory and invasive activity of OS cells. *P < 0.05; **P < 0.01; ***P < 0.001; ****P < 0.0001; Student’s t-test. GFP, green fluorescent protein; sh, short hairpin RNA.

The above RNA-seq results highlighted a potential role for Tspan9 as a regulator of OS cell migration and invasion. To analyze the metastatic role of this gene, highly metastatic HOS and U2OS cells were thus utilized for subsequent assays. We began by knocking down Tspan9 in HOS cells, which expressed high basal levels of this gene, and confirmed stable knockdown therein by fluorescent microscopy, qRT-PCR, and Western blotting (**Figure 4A**). In subsequent woundhealing and Transwell assays, we found that this reduction in Tspan9 expression was associated with significantly impaired migratory activity (**Figures 4B, C**). Similarly, Tspan9 knockdown decreased HOS cell invasivity in a Matrigel-based invasion assay relative to control cells (**Figure 4C**). We additionally overexpressed Tspan9 in U2OS cells which expressed low endogenous Tspan9 levels (**Figure 4D**), and found that the upregulation of this tetraspanin increased tumor cell migratory (**Figures 4E, F**) and invasive (**Figure 4G**) activity. Together, these data suggest that Tspan9 serves as a positive regulator of *in vitro* OS cell metastasis.

### Tspan9 Knockdown Impairs EMT Induction and *In Vivo* OS Cell Metastasis

**Figure 5:**
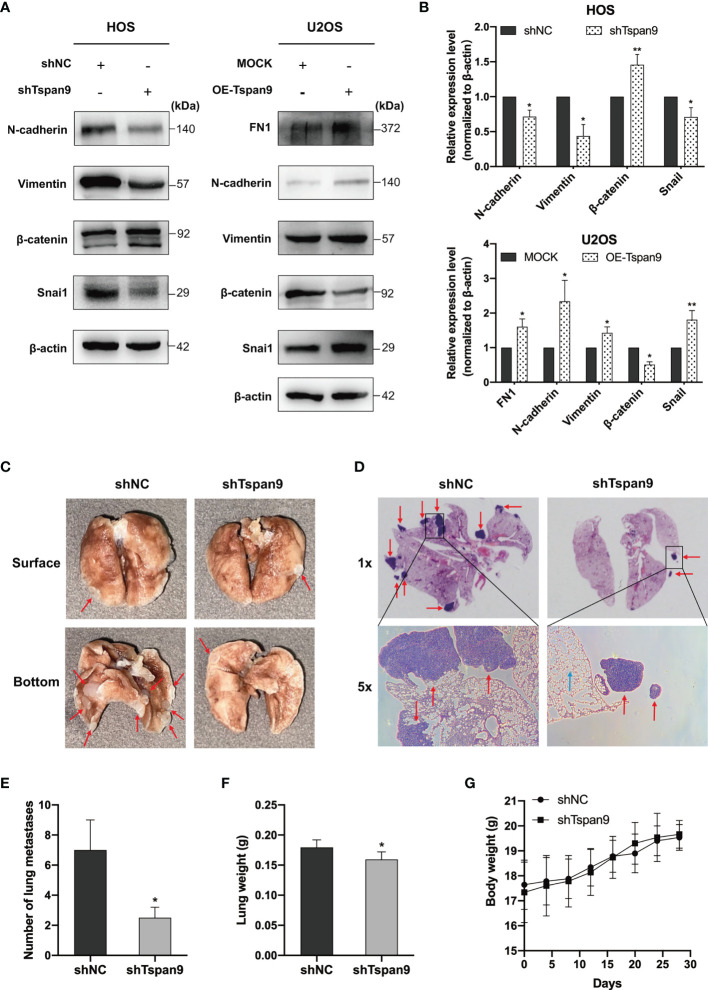
Tspan9 regulates EMT, and knocking down of it impairs *in vivo* OS cell metastasis. **(A)** EMT marker protein levels and associated transcription factor expression were assessed in HOS and U2OS cells stably expressing shTspan9/OE-Tspan9 or control constructs. **(B)** Quantification of the Western blotting results presented in **(A)**. **(C, D)** HOS cells stably expressing shTspan9 or control constructs were injected *via* the lateral tail vein into nude mice to establish a model of pulmonary metastasis (n=5/group). Left: representative lung images; Right: representative H&E staining results (1x and 5x). Pulmonary nodules are indicated by red arrows, while normal alveolar tissue is indicated by blue arrows. **(E)** Numbers of metastatic pulmonary nodules in the indicated groups. **(F)** Lung weights on day 28 post-HOS tumor cell injection. **(G)** Murine body weight was assessed every 4 days. *P < 0.05; **P < 0.01.

The EMT process is an early and essential step in the metastatic process that enables tumor cells to migrate away from the primary tumor site. Several prior reports have demonstrated that tetraspanins are closely linked to EMT initiation (30, 31). Our RNA-seq analysis, notably, identified FN1, which is a mesenchymal EMT marker, as an important regulatory target of Tspan9. FN1 is also involved in certain cancer metastasis-related GO processes such as regulation cell adhesion and extracellular structure organization. Through Western blotting, we found that Tspan9 overexpression was associated with marked FN1 upregulation in these OS tumor cells (**Figures 5A, B**). To test the ability of Tspan9 to control EMT induction in this oncogenic context, we thus analyzed the expression of EMT-related proteins. Western blotting analysis revealed that Tspan9 knockdown was associated with a decrease in the expression of both mesenchymal markers (N-cadherin and Vimentin) and EMT-regulating transcription factor (Snai1) (**Figures 5A, B**), while related to an increasing in epithelial cell marker (β-catenin). In contrast, the expression of mesenchymal markers and Snai1 were elevated while the epithelial marker β-catenin was downregulated after overexpressing Tspan9 in U2OS cells (**Figures 5A, B**). Furthermore, the changes in ZEB and Twist families as EMT transcriptional factors are shown in **Figure S1**. RT-PCR analysis revealed that the mRNA levels of ZEB1, ZEB2 and Twist1 were not affected by Tspan9 knockdown and overexpression.

HOS cells in which Tspan9 had been stably knocked down exhibited impaired migratory and proliferative activity, together with impaired EMT initiation, whereas these changes were reversed when this Tspan9 protein was overexpressed in U2OS cells. To further confirm the ability of Tspan9 to similarly regulate metastatic progression *in vivo*, we injected shNC and shTspan9 HOS cells into the lateral tail vein of female BALB/c nude mice and then assessed pulmonary metastases. Following a 4-week period, 4 mice in the control group developed metastatic lung nodules, whereas they were evident in just 2 mice in the shTspan9 group. Representative lung images are shown in **Figure 5C**, while corresponding H&E-stained tissue sections are shown in **Figure 5D**. Statistical analyses revealed that there were significantly fewer pulmonary metastases in mice that were injected with HOS cells expressing shTspan9 as compared to mice injected with control tumor cells (**Figure 5E**). Lung weight values were also significantly lower in the shTspan9 group relative to the control group (**Figure 5F**), although body weight was comparable between these groups (**Figure 5G**). Together, these results provided robust evidence for the role of Tspan9 as a driver of OS cell metastasis *in vitro* and *in vivo* in part owing to its ability to initiate the EMT process.

### Tspan9 Regulates OS Cell Metastasis Through Interactions With Integrin β1 and the Activation of Downstream FAK-Ras-ERK1/2 Signaling

**Figure 6:**
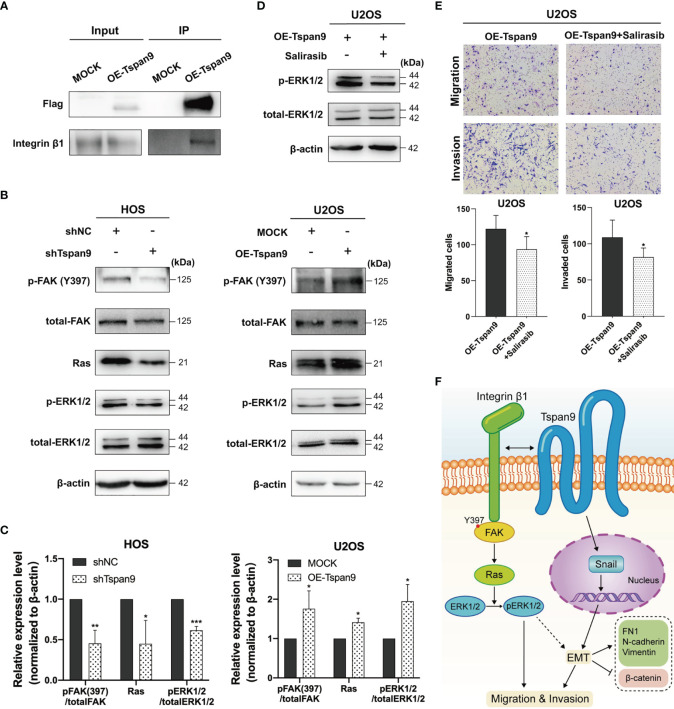
Tspan9-β1 interactions promote FAK-Src-Ras-ERK1/2 pathway signaling and OS metastasis. **(A)** Interactions between Tspan9 and integrin β1 were detected in a co-IP assay. **(B)** FAK-Ras-ERK1/2 pathway proteins (FAK^Y397^, total-FAK, Ras, pERK1/2, and total-ERK1/2) were analyzed *via* western blotting, with β-actin as a loading control. **(C)** Quantification of the Western blotting results presented in **(B)**. **(D)** Western blotting was used to assess Ras downstream signaling in OE-Tspan9 U2OS cells following Salirasib treatment (50μM). **(E)** Following treatment with Salirasib (50μM), OE-Tspan9 cells were analyzed in migration and invasion assays, with representative cells being shown. **(F)** Schematic presentation of mechanism underlying Tspan9-mediated OS metastasisis. All analyzes were repeated two or three times. Data are means ± SD. *P < 0.05; **P < 0.01; ***P < 0.001.

Distant metastases of OS, especially lung metastases, are difficult to control and usually have a poor prognosis. As such, we sought to further understand the mechanisms whereby Tspan9 influences OS cell metastasis at the molecular level. Tetraspanins are known to form TEM complexes with a variety of different proteins at the plasma membrane, including integrins. These tetraspanins further serve as essential regulators of integrin compartmentalization and signal transduction. Through a mass spectrometry approach (**Table S1**) and co-IP analyses (**Figure 6A**), we found that Tspan9 can interact with integrin β1. Integrin β1 binding to collagen triggers intracellular signaling *via* FAK, which becomes autophosphorylated at the Tyr397 or Tyr925 residues to generate SH2-bearing sites capable of further triggering Ras-dependent MAPK signaling (32). To explore the relationship between integrin β1 signaling and Tspan9, we evaluated FAK and ERK1/2 expression and phosphorylation levels, as well as Ras in OS cell lines in which Tspan9 had been knocked down or overexpressed. Western blotting results showed that Tspan9 knockdown in HOS cells was associated with reduced pFAK (Try397), Ras, and pERK1/2 levels as compared to control cells, whereas Tspan9 overexpression in U2OS cells yielded the opposite effect (**Figures 6B, C**). To further determine whether these migratory and invasive changes were linked to Tspan9-β1 complex mediated cell signaling, we treated these cells with a Ras inhibitor (Salirasib) and then analyzed the relative signaling proteins involved in FAK-Ras-ERK1/2 and the metastatic activity of OS cells. This treatment approach resulted in the marked suppression of ERK1/2 phosphorylation downstream of Ras (**Figure 6D**). Ras inhibitor treatment also decreased OS cell migration and invasion relative to that observed for control cells (**Figure 6E**). In addition, we established stable β1 integrin-knockdown cells by shRNA in Tspan9-overexpression U2OS cells. Compared with shControl, shIntegrin β1 resulted in a reduce in the expression of Ras (**Figure S2A**). Also, highly invasive OE-Tspan9 U2OS cells stably down-regulating integrin β1 exhibited impaired capacity of migration and invasion as compared with their counterparts (**Figure S2B**). Together, these data indicate that Tspan9 at least partially promotes OS cell invasive and migratory activity *via* binding to integrin β1 integrin and thereby inducing integrin β1 mediated downstream FAK-Ras-ERK1/2 signaling (**Figure 6F**).

## Discussion

Tetraspanin-9 is a 27 kD protein encoded on chromosome 12p13.33-p13.32 in humans, and is expressed at high levels in renal, cardiac, and placental tissues. Recent studies of Tspan9 have highlighted its function as a suppressor of migratory, invasive, and autophagic activity in gastric cancer cells. The mechanisms whereby Tspan9 achieves these regulatory activities, however, have yet to be defined, particularly in other tumor types. This study is the first to have clarified the functional role of Tspan9 in the context of OS development.

Herein, we evaluated the expression of Tspan9 in OS cell lines and tissues and found it to be significantly upregulated in both analyses of extant GEO datasets. When Tspan9 expression was knocked down in OS cells, this suppressed their viability and proliferation, suggesting that Tspan9 plays an oncogenic role distinct from its function as a tumor suppressor in gastric cancer. We subsequently generated HOS and U2OS cell lines in which Tspan9 was stably knocked down or overexpressed, respectively, and further confirmed that the stable knockdown of this tetraspanin inhibited OS cell migration and invasion *in vitro* and *in vivo*. Conversely, the overexpression of Tspan9 enhanced the metastasis of these cells, confirming its carcinogenic role in this cancer type.

To better explore the role of Tspan9 as a regulator of OS onset and progression, we conducted an RNA-seq analysis in which we identified 96 and 115 genes that were respectively up- and downregulated in cells in which Tspan9 was stably knocked down. FN1 was notably detected among these downregulated DEGs, and is associated with GO terms including regulating cell adhesin, extracellular structure organization, and cell-matrix adhesion, suggesting that Tspan9 may play an important role as a regulator of OS cell migratory and invasive activity. In addition, FN1 serves as an important biomarker associated with EMT induction that is known to influence adhesion, invasion, and migration (33, 34). Metastatic progression is a primary cause of death among OS patients, and EMT induction is central to the initiation of invasive pro-metastatic activity. During the EMT process, epithelial-like cells acquire mesenchymal characteristics including increased motility and reductions in cell-cell adhesion (35). A growing body of evidence suggests that many tetraspanins can shape oncogenic processes through the suppression or induction of the EMT process. For example, Qi et al. (14) reported that the knockdown of Tspan9 in gastric cancer cells led to increases in the levels of N-cadherin, Vimentin, Twist, and ZEB1 in these cells, together with decreased E-cadherin expression, thus suggesting that Tspan9 regulated gastric cancer cell metastasis in part *via* modulating EMT induction. CD82 is a tetraspanin that, when expressed at low levels and in the context of elevated Vimentin expression, was associated with a worse lung cancer patient prognosis (36). Lee et al. (37) also found that CD82 was able to suppress prostate cancer cell EMT induction and metastasis *via* disrupting TGF-β1/Smad and Wnt/β-catenin signaling activity. Other tetraspanin proteins including CD63, CD73, CD151, and Tspan8 have also been shown to modulate the EMT process and tumor progression in colorectal cancer, gastric cancer, melanoma, and renal cell carcinoma (38–41). In light of the above findings, we further analyzed FN1 expression in our experimental model system and found FN1 to be upregulated following Tspan9 overexpression in U2OS cells, with these changes also correlated with the increased expression of EMT markers and transcription factors including N-cadherin, Vimentin, and Snai1. In contrast, the opposite findings were observed upon Tspan9 knockdown. Together, our findings thus suggest that Tspan9 can promote the migratory and invasive activity of OS cells at least in part by promoting EMT induction.

Tetraspanins exhibit a unique ability to form TEM membrane structures wherein they interact with specific enzymes and/or receptors to influence intracellular signaling activity *via* the control of the functionality or trafficking of these molecules and the facilitation of interactions between specific molecules (42). Herein, we explored the relationship between key integrins known to promote OS cell metastasis and Tspan9. Li et al. (25) previously demonstrated the ability of integrin β1 to inhibit OS cell apoptosis and to thus enhance the migratory activity of these cells, while Jiang et al. (24) further observed increased EMT induction and invasive activity in OS cells upon integrin β1 upregulation. Ren et al. (43) found that the αvβ3 integrin was able to induce ERK1/2 signaling in OS cells to promote their invasion and migration, and specific interactions between tetraspanin proteins and this integrin have been shown to govern tumor metastasis (44). These prior findings suggest that integrins can serve to stimulate OS cell metastasis *via* ERK signaling and/or EMT induction. Interactions between particular tetraspanin proteins and integrin β1 have also been explored, as in the case of CD82, which can suppress tumor metastasis by interacting with integrin β1 in TEM domains and thereby disrupting the ability of this integrin to interact with the fibronectin matrix, impairing associated intracellular signaling (45, 46). In contrast, the migration and invasion of glioma cells are enhanced by the CD151-α3β1 complex, which promotes FAK^Y397^ activation and associated GTPase signaling (47). Herein, we confirmed that Tspan9 was able to directly interact with integrin β1 through mass spectrometry and co-IP analyses, while we further identified a role for Tspan9 as a regulator of ERK1/2 pathway activation through RNA-seq assays. The recruitment of integrin β1 to the ECM promotes FAK^Y397^ autophosphorylation, in turn leading to the recruitment of Src family kinases and the consequent phosphorylation of other FAK residues including Try925, thereby triggering downstream Ras-ERK signaling (32, 48, 49). Lee et al. determined that CD82 can interact with the α3β1/α5β1 integrins in prostate cancer cells, ultimately impairing EMT induction by inhibiting FAK/Src signaling (46). Wang et al. further demonstrated that in cholangiocarcinoma cells, Tspan1 can drive the EMT process through interactions with integrin α6β1 and the consequent amplification of downstream signaling (31). The Ras-ERK pathway and associated downstream factors are closely tied to the EMT (50, 51). Interactions between Tspan9 and integrin β1 may thus strongly impact the ability of OS cells to proliferate and metastasize by shaping intracellular signaling activity. Herein we evaluated FAK and ERK1/2 phosphorylation, and found that these, together with Ras expression, were positively correlated with Tspan9 expression. The Ras inhibitor Salirasib was also able to inhibit Tspan9 overexpression-induced ERK1/2 phosphorylation, migration, and invasion in OS cells. Furthermore, compared with highly invasive Tspan9-overexpression group, stable integrin β1-knockdown in OE-Tspan9 U2OS cells showed inhibited Ras expression, reduced migration and invasion ability. As such, Tspan9 may promote OS cell metastatic progression by binding to integrin β1 and thereby promoting FAK-Ras-ERK1/2 signaling activity, thus leading to EMT induction. However, further research will be necessary to explore the mechanisms whereby Tspan9 modulates this EMT process in OS cells.

## Conclusion

We herein found for the first time that Tspan9 can function to promote human OS progression. Both OS tumor tissue samples and cell lines exhibited marked Tspan9 upregulation, and the overexpression of this gene was associated with enhanced proliferation, EMT induction, and metastasis in OS cells through a mechanism whereby Tspan9 interacts with integrin β1 to augment downstream integrin-mediated FAK-Ras-ERK1/2 signaling. Together, our results provide a firm foundation for future analyses of the specific mechanisms governing OS progression and may highlight novel approaches to improving OS patient therapeutic outcomes.

## Data Availability Statement

The original contributions presented in the study are included in the article/**Supplementary Material**. Further inquiries can be directed to the corresponding authors.

## Ethics Statement

The animal study was reviewed and approved by Ethics Committee of the Third Affiliated Hospital of Soochow University (approval number: 2021-007).

## Author Contributions

YW and LP conceptualized the study. JSW contributed to the data curation. LG conducted the formal analysis. SS, JWW, and LW investigated the study. LT contributed to the methodology. XY and XH conducted the project administration. SF and JZ conducted the visualization. SS wrote the original draft. SS and LP wrote, reviewed, and edited the manuscript. All authors contributed to the article and approved the submitted version.

## Funding

The present study was supported by the National Natural Science Foundation of China (grant No. 81903661), and the Project of State Key Laboratory of Radiation Medicine and Protection of Soochow University (grant No. GZK1202129).

## Conflict of Interest

The authors declare that the research was conducted in the absence of any commercial or financial relationships that could be construed as a potential conflict of interest.

## Publisher’s Note

All claims expressed in this article are solely those of the authors and do not necessarily represent those of their affiliated organizations, or those of the publisher, the editors and the reviewers. Any product that may be evaluated in this article, or claim that may be made by its manufacturer, is not guaranteed or endorsed by the publisher.
