# Supplementary material for: Tspan9 Induces EMT and Promotes Osteosarcoma Metastasis via Activating FAK-Ras-ERK1/2 Pathway
Source: Front Oncol. 2022 Feb 23;12:774988. doi: 10.3389/fonc.2022.774988 (PMC8906905; doi:10.3389/fonc.2022.774988)
Supplement: Supplementary file 1 [file Image_1.pdf]

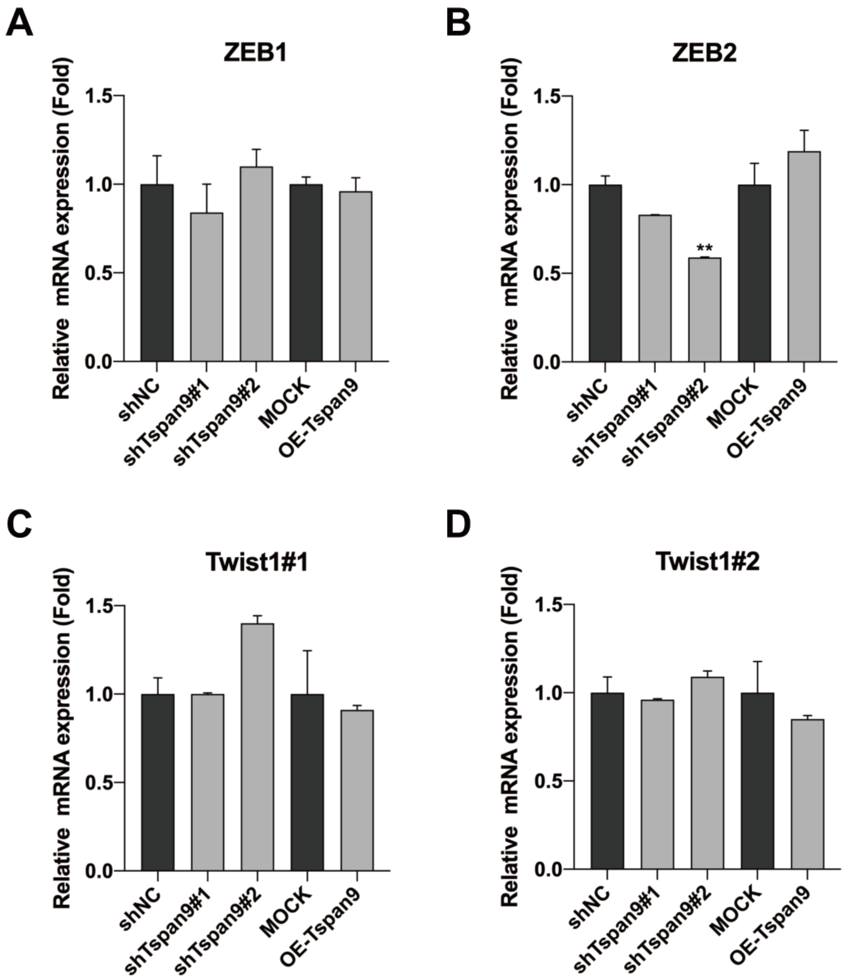


**Figure S1.** Relative changes of ZEB and Twist families following knockdown and overexpression of Tspan9 in OS cells were examined using qRT-PCR. **(A-D)** No significant alterations in the mRNA levels of ZEB1, ZEB2 and Twist1 were observed. ZEB, zincfinger E-box binding homeobox; Twist, twist family bHLH transcription factor.
