# Supplementary material for: Tspan9 Induces EMT and Promotes Osteosarcoma Metastasis via Activating FAK-Ras-ERK1/2 Pathway
Source: Front Oncol. 2022 Feb 23;12:774988. doi: 10.3389/fonc.2022.774988 (PMC8906905; doi:10.3389/fonc.2022.774988)
Supplement: Supplementary file 2 [file Image_2.pdf]

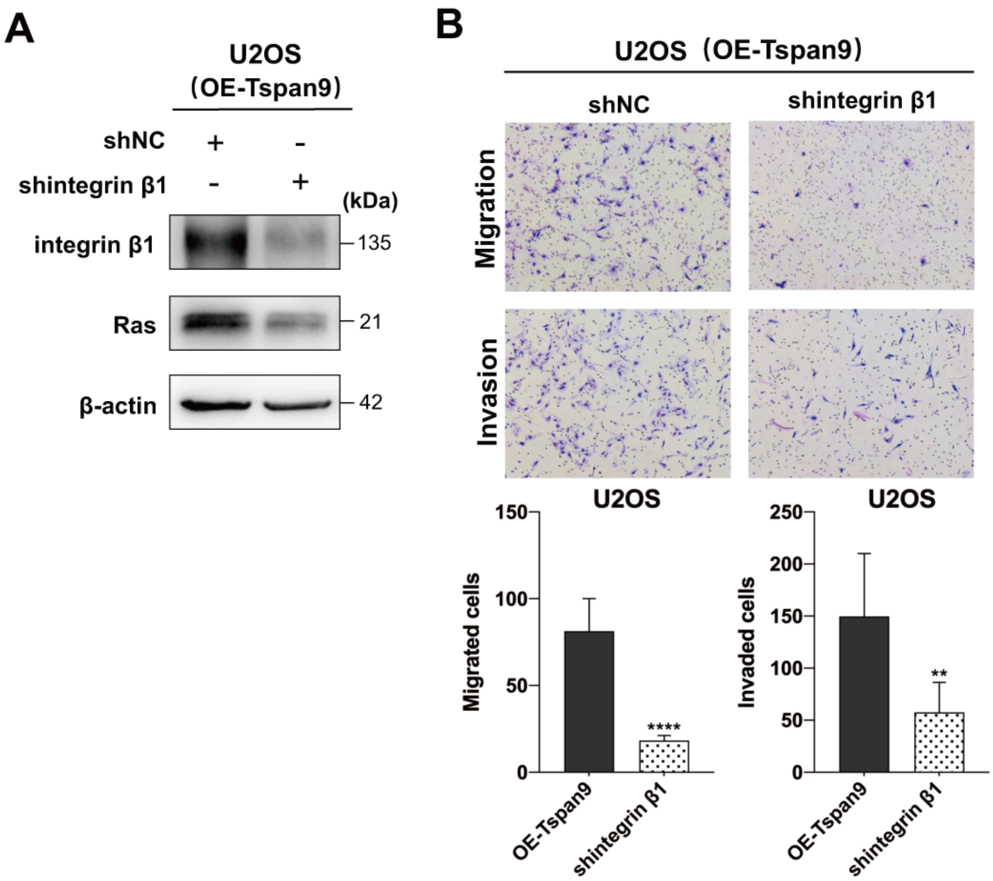

Figure S2. Knockdown of β1 integrin inhibits downstream Ras expression and the metastasis of OS cells in vitro. **(A)** Knockdown efficiency of integrin β1 in Tspan9-overexpression U2OS cells and regulation of downstream Ras were confirmed by Western blotting. **(B)** The impact of integrin β1 knockdown on OS cell migration and invasion was assessed via a Transwell approach. All analyzes were repeated two or three times. Data are means ± SD. ***P* < 0.01; *****P* < 0.0001.
