# Supplementary material for: Tspan9 Induces EMT and Promotes Osteosarcoma Metastasis via Activating FAK-Ras-ERK1/2 Pathway
Source: Front Oncol. 2022 Feb 23;12:774988. doi: 10.3389/fonc.2022.774988 (PMC8906905; doi:10.3389/fonc.2022.774988)
Supplement: Supplementary file 4 [file Table_2.docx]

**Tables**

**Table S2.** The sequences information of qRT-PCR primers and shRNAs.

| **Name** |  | **Sequences** |
| --- | --- | --- |
| Twist1#1 | Forward | ACGCTGCCCTCGGACAA |
|  | Reverse | CCTGGTAGAGGAAGTCGATGTACCT |
| Twist1#2 | Forward | GCGCTGCGGAAGATCATC |
|  | Reverse | GGTCTGAATCTTGCTCAGCTTGT |
| ZEB1 | Forward | CACCATCCCCATCACCTCTAA |
|  | Reverse | GCACCCTCAGCTGTGTACAAGT |
| ZEB2 | Forward | TAAGGGAGGGTGGAGTGGAA |
|  | Reverse | CTGGACCATCTACAGAGGCTTGT |
| Integrin β1 shRNA |  |  |
| shIntegrin β1 | 5’-3’ | GACTGAGCATGATGAAAGTTTCAA  GAGAACTITCATCATGCTCAGTCTT |
| Negative Control (shNC) | 5’-3’ | GTTCTCCGAACGTGTCACGTTTCAAG  AGAACGTGACACGTTCGGAGAACTT |
